# Addition by Subtraction: A Novel Device for Pulmonary Embolus Thrombectomy

**DOI:** 10.1016/j.jscai.2024.102508

**Published:** 2024-12-28

**Authors:** Joseph M. Kim, Eric A. Secemsky

**Affiliations:** a Richard A. and Susan F. Smith Center for Outcomes Research, Beth Israel Deaconess Medical Center, Boston, Massachusetts; b Division of Cardiovascular Medicine, Beth Israel Deaconess Medical Center, Harvard Medical School, Boston, Massachusetts

**Keywords:** catheter-directed therapy, pulmonary embolism, thrombectomy

The field of acute pulmonary embolus (PE) treatment has seen the rapid adoption of catheter-based interventions over the past decade.^1^ Although systemic anticoagulation remains the cornerstone of therapy for acute PE, minimally invasive devices designed to pharmaco-mechanically recanalize the pulmonary arteries have helped fill a niche for those too sick to respond to systemic anticoagulation alone but with the goal of avoiding the risks of systemic thrombolysis or surgery. One class of catheter-based devices is mechanical aspiration thrombectomy (MT), designed to directly evacuate thrombus from the pulmonary arteries (Table 1). Devices in this class already approved for the treatment of PE include the FlowTriever system (Inari Medical) and the Indigo system (Penumbra Inc).^1^, ^2^, ^3^

**Table 1:** Catheter-directed mechanical thrombectomy devices approved in the United States.

Device	Mechanism of action	Procedural considerations	IDE trial name	Primary efficacy end point	Primary safety end point
AngioVac system (AngioDynamics, Inc)	Large-bore cannula with funnel-shaped inflow tip to engage and aspirate thromboembolus	26F access for inflow, 16F-20F access for outflow; requires VV-ECMO circuit and perfusion team	–	–	–
Approved for PE thrombectomy					
FlowTriever system (Inari Medical)	Large-bore cannula to engage and aspirate thromboembolus; adjunctive nitinol disks can be used to assist with clot engagement and retrieval	20F-24F access; significant collateral blood loss expected during aspiration	FLARE (NCT02692586)	Mean RV/LV ratio reduction in 48 hours: 1.53-1.15	At 48-hour postprocedure^a^: 1 hemoptysis, 1 clinical deterioration, 1 cardiogenic shock, 1 ventricular fibrillation, 1 death
Indigo system (Penumbra Inc)	Relatively smaller bore cannula to engage thromboembolus; continuous aspiration	12F-16F access; larger clots can be difficult to aspirate	EXTRACT-PE (NCT03218566)	Mean RV/LV ratio reduction in 48 hours: 1.47-1.04	At 48-hours postprocedure^b^: 2 major bleeding, 1 device-related clinical deterioration, 1 device-related pulmonary vascular injury, 1 device-related death
AlphaVac F18^85^ system (AngioDynamics, Inc)	Large-bore cannula with funnel-shaped inflow tip to engage and aspirate thromboembolus	18F access; does not require VV-ECMO circuit or perfusion team	APEX-AV (NCT05318092)	Mean RV/LV ratio reduction in 48 hours: 1.51-1.07	At 48-hours postprocedure^b^: 3 major bleeding, 1 hypovolemic shock, 1 pulmonary vascular injury and clinical deterioration, 0 deaths

IDE, investigational device exemption; LV, left ventricle; PE, pulmonary embolus; RV, right ventricle; VV-ECMO, veno-venous extracorporeal membrane oxygenation.

a Valve Academic Research Consortium (VARC-2) definition of major bleeding.

b Global Utilization of Streptokinase and Tissue Plasminogen Activation for Occluded Coronary Arteries (GUSTO) definition of major bleeding.

Although significant progress has been made in the use of MT, there remain risks associated with this approach. First, due to the large-bore cannulas required, access-site complications such as bleeding are real. In addition, directly engaging the clot increases the risk of dislodging emboli further into the pulmonary vasculature, and the stiffer character of the catheters can cause trauma within the cardio-pulmonary system. Finally, there can be significant collateral blood loss during efforts to aspirate the clot.^1^

One of the earliest thrombectomy devices used in the cardio-pulmonary system was the AngioVac system (AngioDynamics, Inc). Though not specifically approved for aspiration of PE, the AngioVac device uses a 26F inflow access catheter with a funnel-shaped tip to directly engage and aspirate thrombi; however, with its bulky size and design for intracardiac and large vessel pathology, this was not the ideal cannula to deliver to the pulmonary artery. In addition, the device necessitates connecting the patient to a veno-venous extracorporeal membrane oxygenation (V-V ECMO) circuit during use.^1^ This can be time-consuming and resource-heavy, and there has been a need for a more pragmatic device for the aspiration of acute PE.

In this issue of *JSCAI*, the authors present the results of the APEX-AV investigational device exemption (IDE) trial (NCT05318092) evaluating the safety and efficacy of the new AlphaVac F18^85^ MT system (AngioDynamics, Inc).^4^ The AlphaVac System features a vacuum handle and an 18F cannula with an expandable funnel tip that is expandable to 33F to prevent clogging during the removal of large material. Notably, the AlphaVac system does not require V-V ECMO and can be used quickly in any procedural suit.

The APEX-AV IDE trial evaluated the AlphaVac F18^85^ system through a prospective, single-arm, multicenter study that enrolled patients with acute intermediate-risk PE. The trial enrolled 122 patients with confirmed acute PE, a right ventricle (RV)/left ventricular (LV) diameter ratio of ≥0.9 on computed tomography angiography, and hemodynamic stability. The primary efficacy end point was the reduction in RV/LV diameter ratio at 48 hours, whereas the primary safety end point was the rate of major adverse events within 48 hours, including major bleeding, device-related deaths, and pulmonary or cardiac injuries. Secondary end points included reductions in pulmonary artery pressure, clot burden (measured via the modified Miller index), and lengths of intensive care unit and hospital stays.

The mean RV/LV ratio decreased significantly by –0.45 ± 0.27 at 48 hours (*P* < .001), exceeding the predefined goal of –0.12. Clot burden was reduced by 35.5% on the modified Miller index, and mean pulmonary artery pressure declined from 27.8 mm Hg to 21.8 mm Hg (*P* < .001). The major adverse event rate was 4.1%, below the safety threshold of 25%, with most adverse events attributed to access-site bleeding. No deaths or unanticipated device complications were reported. These findings affirmed the safety and efficacy of the AlphaVac system for treating intermediate-risk PE.

The most significant addition of the AlphaVac system is in its subtraction—it retains the features of the AngioVac system without the need to connect the patient to the V-V ECMO circuit. The authors also highlight that the procedural time of the AlphaVac system was shorter than that reported for the FlowTriever device in the FLARE trial (37.2 ± 17.7 minutes vs 57.7 ± 29.6 minutes), suggesting greater procedural efficiency, although this can only be verified in a head-to-head trial.^2^ One particular feature of the AlphaVac device is the volume-limiting switch on the handle designed to minimize blood loss during the vacuum-assisted aspiration, which resulted in ≤250 cc blood loss in 62.3% of cases. Despite this feature, it must be noted that 4 patients developed device-related severe adverse events, all involving bleeding/hemorrhage.

The APEX-AV trial was a well-run IDE trial that cleared the path for approval of the AlphaVac device by the United States Food and Drug Administration via 510(k) clearance in April 2024.^5^ This is now the third thrombectomy device with a PE indication, providing a more robust armamentarium of catheter-based systems for treating patients with acute PE (Table 1). Looking forward, it will be crucial to continue the evaluation with clinical outcomes associated with the use of the device, ideally in comparison to other competing systems. The recently published PEERLESS trial (NCT05111613) compared the FlowTriever system to catheter-directed thrombolysis, providing a new benchmark of head-to-head device comparisons that can advance treatment algorithms.^6^ In addition, end points such as the RV/LV ratio used in the APEX-AV trial are required for regulatory approval and are based on older observational data suggesting risk associated with abnormal ratios; however, no prospective, randomized data have supported the assumption that faster reduction of the RV/LV ratio results in reduced mortality.^7^ As more catheter-based therapies for PE enter the market, it will be imperative for clinicians to have randomized evidence that these devices outperform anticoagulation alone with clinical end points. Several trials, such as PEERLESS II (NCT06055920), HI-PEITHO (NCT04790370), PE-TRACT (NCT05591118), and STORM-PE (NCT05684796), are now underway and will inform how each device impacts mortality, escalation of critical care interventions, and functional outcomes.^8^, ^9^, ^10^, ^11^

The APEX-AV trial demonstrated that the AlphaVac F18^85^ system can be an important addition to the arsenal of therapies for PE. We welcome the unique features and pragmatic design that can advance acute PE care. Further investigations of the device will determine how this system fits into the rapidly evolving ecosystem of catheter-based PE treatment options.
